# High-flow nasal cannula and intubation risk in severe PjP: methodological and clinical perspectives

**DOI:** 10.1186/s13613-025-01581-6

**Published:** 2025-11-17

**Authors:** Lulu Wang, Jinying Cheng

**Affiliations:** 1 Department of Critical Care Medicine, Binzhou Medical University Hospital, Binzhou, Shandong 256603 China; 2 Department of Infectious Diseases, Binzhou Medical University Hospital, Binzhou, Shandong 256603 China

To the Editor,

We read with great interest the article by Reizine et al. [1], which analyzed 248 ICU patients with Pneumocystis jirovecii pneumonia (PjP) from the PRONOCYSTIS cohort over 2011–2021, comparing initial high-flow nasal cannula (HFNC), standard oxygen (SO), and non-invasive ventilation (NIV). HFNC was associated with a lower intubation rate (IPTW-weighted HR 0.41, 95% CI 0.24–0.69), though no day-90 survival benefit was observed. We commend the authors for addressing an important knowledge gap in respiratory management of immunocompromised patients [2].

First, the primary endpoint analysis censored patients who died without intubation, potentially overestimating the“intubation-free”probability. A competing-risks approach (e.g., Fine–Gray [3]) could yield more robust estimates. In addition, grouping patients who received alternating HFNC and NIV under “NIV” may have diluted potential differences; a time-updated or multi-state model could address treatment sequencing.

Second, Given the 10-year, multicenter design, practice evolution and inter-center variability may confound results. Sensitivity analyses incorporating “center” and “epoch” effects are encouraged. The use of the 4% FiO₂ estimation formula [4] in SO patients could introduce systematic bias in PaO₂/FiO₂ calculation; SpO₂/FiO₂–based sensitivity analysis may help validate findings.

Third, Long-term corticosteroid therapy, solid tumor, and higher SOFA score independently predicted mortality. Exploring whether HFNC’s intubation-reduction effect persists in these high-risk subgroups (especially HIV-negative malignancy or transplant recipients) would enhance clinical targeting.

Fourth, only 11.3% of patients had received anti-Pneumocystis prophylaxis, underscoring a missed preventive opportunity. Coupling optimized prophylaxis with standardized HFNC escalation protocols (e.g., ROX index thresholds [5], early awake prone positioning, predefined intubation criteria) may improve outcomes and safety.

In conclusion, this study provides important real-world evidence supporting HFNC to reduce intubation in critically ill PjP, though survival benefit remains unproven. Future prospective studies addressing competing risks, treatment sequencing, and heterogeneity are warranted. We congratulate the authors on this valuable contribution and look forward to further research in this field.

## Data Availability

Not applicable.
